# Severe Hepatic Iron Overload and Cirrhosis in an HFE C282Y Heterozygote With Autoimmune Hepatitis: A Case of Genotype-Phenotype Discordance

**DOI:** 10.7759/cureus.105851

**Published:** 2026-03-25

**Authors:** Rodrigo Furlan Silva Fabri, Rodolfo Myronn De Melo Rodrigues, Deiptan Prabakar

**Affiliations:** 1 Internal Medicine, Texas Tech University Health Sciences Center El Paso Paul L. Foster School of Medicine, El Paso, USA

**Keywords:** autoimmune hepatitis, chronic liver disease, cirrhosis, genotype–phenotype discordance, hereditary hemochromatosis, hfe c282y heterozygosity, hyperferritinemia, iron overload, therapeutic phlebotomy

## Abstract

Hereditary hemochromatosis is a disorder of iron metabolism characterized by increased intestinal iron absorption and progressive parenchymal iron deposition, most commonly associated with homozygous mutations in the HFE gene. In contrast, heterozygous carriers of HFE mutations are generally considered to have low clinical penetrance and are not expected to develop clinically significant iron overload or advanced liver disease. However, iron homeostasis may be substantially altered in the presence of chronic liver inflammation and immune-mediated hepatic injury. We present the case of a woman in her early 60s with a heterozygous HFE C282Y mutation who developed marked hyperferritinemia, biopsy-confirmed hepatic iron overload, and established cirrhosis in the setting of autoimmune hepatitis treated with mycophenolate mofetil. Her clinical course was notable for wide fluctuations in ferritin levels over several years, with a peak exceeding 3,800 ng/mL, and subsequent sustained reduction following therapeutic phlebotomy. Management was complicated by anemia, requiring individualized phlebotomy thresholds and transition to a maintenance strategy. This case highlights the diagnostic challenges of interpreting iron indices in patients with inflammatory liver disease and demonstrates that clinically significant iron overload may occur in HFE heterozygotes when additional disease modifiers are present. It underscores the importance of histologic confirmation of iron overload in cases of genotype-phenotype discordance and reinforces the need for individualized management strategies in complex iron-loading conditions.

## Introduction

Hereditary hemochromatosis (HH) is one of the most common inherited metabolic disorders in populations of Northern European ancestry and is characterized by excessive intestinal iron absorption leading to progressive iron deposition in parenchymal organs, particularly the liver [1]. If untreated, iron overload may result in cirrhosis, portal hypertension, hepatocellular carcinoma, diabetes mellitus, cardiomyopathy, and arthropathy [1].

The majority of clinically significant cases of HH are associated with homozygosity for the HFE C282Y mutation, while compound heterozygosity for C282Y and H63D also confers increased risk [2]. In contrast, individuals who are heterozygous for the C282Y mutation are traditionally regarded as asymptomatic carriers with minimal risk of developing clinically meaningful iron overload or end-organ damage [2,3]. Population-based studies have demonstrated low penetrance among heterozygotes, and current guidelines generally do not recommend routine treatment in the absence of additional risk factors [3].

Interpretation of iron indices is particularly challenging in patients with inflammatory or autoimmune conditions. Ferritin, the most widely used surrogate marker of iron stores, is also an acute-phase reactant and may be elevated in chronic inflammation, infection, or liver disease independent of total body iron burden [4]. Dysregulation of the hepcidin-ferroportin axis in chronic liver disease further complicates iron metabolism, potentially leading to increased iron absorption and hepatic deposition even in individuals without high-risk genotypes [5,6].

Autoimmune hepatitis (AIH) is a chronic immune-mediated inflammatory liver disease characterized by interface hepatitis and progressive fibrosis, often requiring long-term immunosuppression [7]. Despite treatment, AIH may progress to cirrhosis, particularly in patients with ongoing inflammatory activity [7]. The coexistence of AIH and iron overload raises important questions regarding the interaction between immune-mediated hepatic injury and iron metabolism. Here, we report a case of severe hepatic iron overload and cirrhosis in a patient heterozygous for the HFE C282Y mutation in the setting of autoimmune hepatitis, illustrating a clear genotype-phenotype discordance and emphasizing the importance of histologic confirmation of iron overload in complex clinical scenarios.

## Case presentation

A 62-year-old woman with a medical history significant for systemic lupus erythematosus (SLE) with prior lupus nephritis in remission, autoimmune hepatitis managed with mycophenolate mofetil, hypertension, and type 2 diabetes mellitus was referred for evaluation and longitudinal management of iron overload and chronic liver disease. She denied alcohol consumption, tobacco use, or illicit drug use. There was no known family history of hereditary iron disorders or chronic liver disease. Evaluation for alternative etiologies of chronic liver disease was unrevealing, including negative viral hepatitis testing.

**Figure 1:**
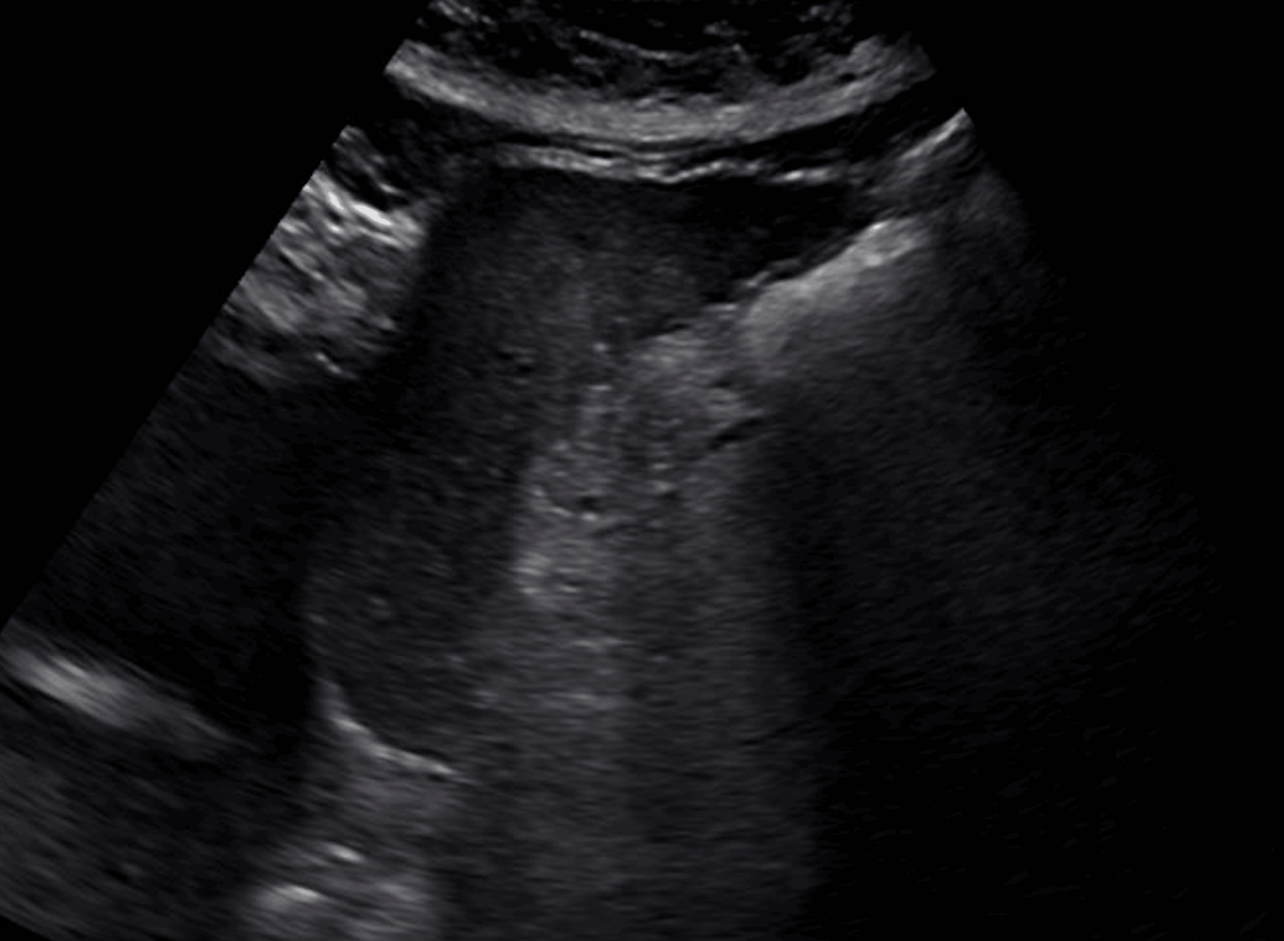
Abdominal Ultrasound Demonstrating Cirrhotic Liver Morphology Abdominal ultrasound image demonstrating heterogeneous hepatic echotexture with coarse parenchymal pattern and irregular liver surface contour, findings consistent with cirrhotic liver morphology.

In 2016, she underwent evaluation for persistently abnormal liver enzymes and elevated iron indices. Genetic testing revealed heterozygosity for the HFE C282Y mutation. Initial abdominal ultrasound demonstrated cirrhotic liver morphology (Figure 1).

**Figure 2:**
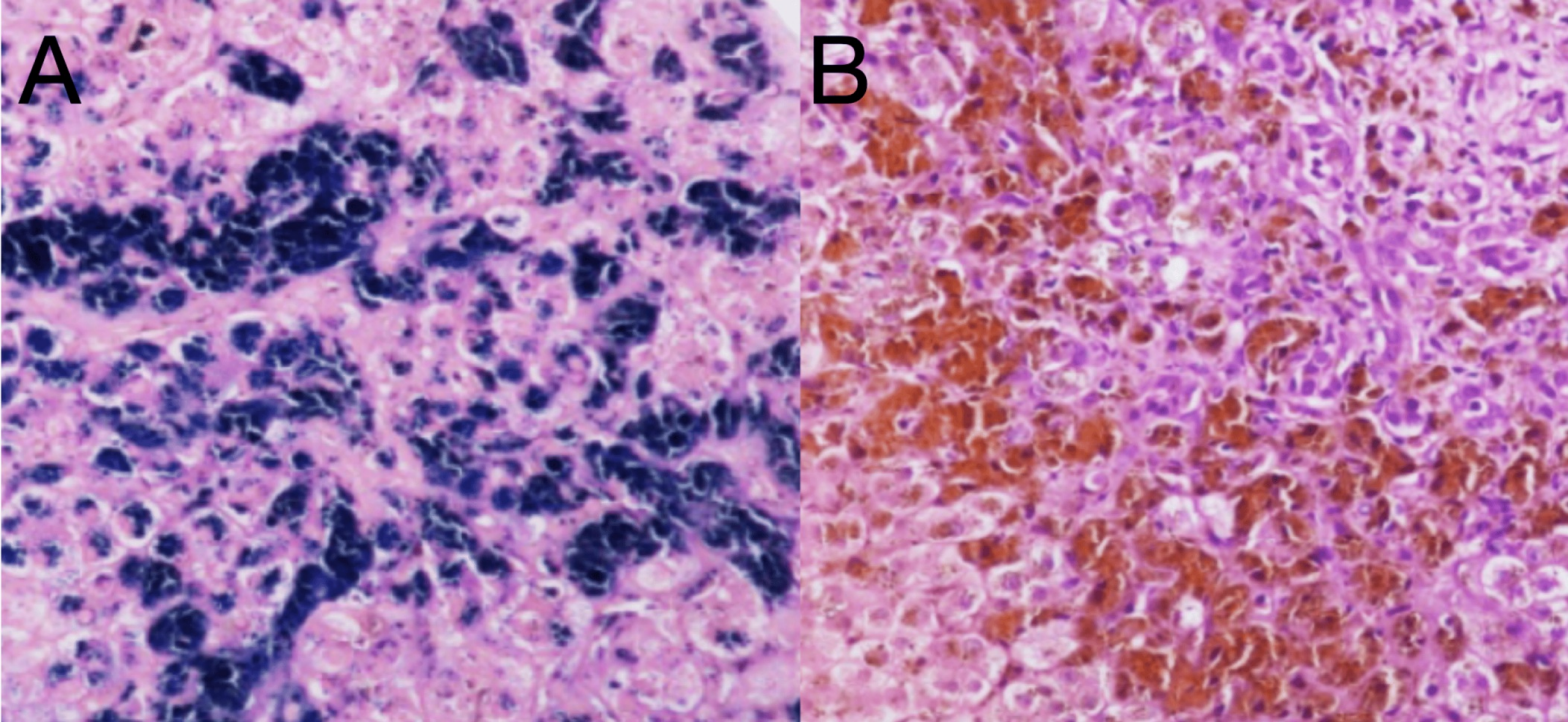
A, Liver Biopsy With Iron stain (Prussian Blue); B, Hematoxylin and Eosin Stain (A) Iron stain (Prussian blue) highlighting abundant iron deposition within hepatocytes and Kupffer cells, appearing as coarse blue granular pigment. (B) Hematoxylin and eosin staining showing distorted hepatic architecture with cirrhotic features and widespread brown granular pigment consistent with hemosiderin accumulation.

Given the discordance between her genotype and degree of biochemical iron overload, a liver biopsy was performed. Histopathologic examination confirmed established cirrhosis with nodular architecture and portal/interface hepatitis consistent with autoimmune hepatitis. Iron staining revealed moderate-to-marked (3+) iron deposition within hepatocytes and Kupffer cells, confirming true parenchymal hepatic iron overload rather than isolated inflammatory hyperferritinemia (Figure 2).

Over the subsequent years, the patient’s serum ferritin levels demonstrated marked variability, ranging from near-normal values to severe hyperferritinemia, with a peak ferritin level of 3,865 ng/mL (reference range: 15-150 ng/mL for adult females). Given histologic confirmation of iron overload, therapeutic phlebotomy was initiated intermittently based on ferritin levels and hematologic tolerance. A clear downward trend in ferritin levels was observed following initiation and resumption of phlebotomy, despite intermittent treatment interruptions due to anemia. The longitudinal ferritin trajectory before and after therapeutic phlebotomy, spanning from 2016 through 2025, is illustrated in Table 1.

**Table 1:** Longitudinal Ferritin Trend Before and After Therapeutic Phlebotomy Serum ferritin levels from 2016 to 2026 demonstrating marked hyperferritinemia at presentation followed by sustained reduction after initiation of therapeutic phlebotomy.

Year	Date/Period	Ferritin (ng/mL)	Therapeutic Phlebotomy	Clinical Interpretation
2016	Initial presentation	3,865	Initiated	Severe hyperferritinemia at diagnosis
2017	Induction phase	2,500	Yes	Persistent iron overload
2018	Induction phase	1,200	Yes	Progressive reduction with phlebotomy
2019	End of induction	450	Yes	Significant biochemical improvement
Jan 2023	Follow-up	206	No	Mild hyperferritinemia
Oct 2023	Follow-up	191	No	Stable iron levels
Jan 2024	Follow-up	259	No	Re-elevation
May 2024	Follow-up	151	No	Partial spontaneous decline
Aug 2024	Follow-up	190	Yes	Phlebotomy resumed
Sep 2024	Follow-up	88.6	Yes	Rapid response
Oct 2024	Follow-up	36.4	No	Ferritin normalization
Nov 2024	Follow-up	39.6	No	Stable
Jan 2025	Follow-up	56.9	No	Maintenance phase
Apr 2025	Follow-up	65.3	No	Stable
Late 2025	Surveillance	164	No	Gradual re-elevation
Early 2026	Surveillance	256	No	Ongoing monitoring

**Figure 3:**
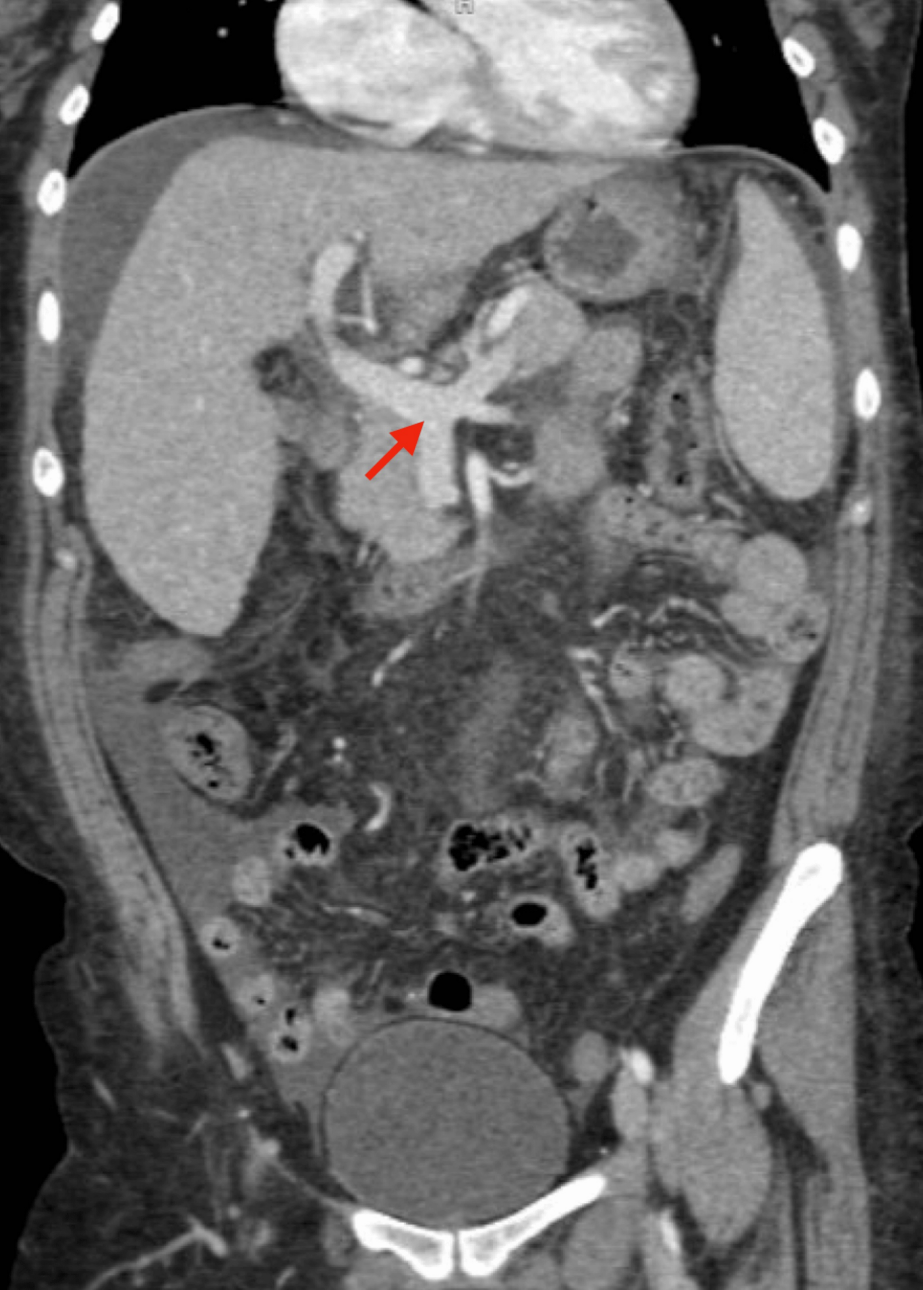
Contrast-Enhanced CT Abdomen Showing Portal Hypertension and Cirrhotic Changes Coronal contrast-enhanced CT image of the abdomen demonstrating findings consistent with portal hypertension. The red arrow indicates abnormal portal venous anatomy at the porta hepatis. The liver parenchyma appears heterogeneous in attenuation, and the spleen demonstrates increased size and differing density. Overall imaging features are compatible with cirrhotic liver morphology in the setting of heterozygous hemochromatosis.

Screening upper endoscopy performed in 2023 in the setting of cirrhosis and portal hypertension demonstrated gastritis without evidence of active gastrointestinal bleeding. Cross-sectional abdominal imaging revealed cirrhotic liver morphology with features of portal hypertension and a small hepatic lesion without radiographic characteristics suspicious for hepatocellular carcinoma. Ongoing imaging surveillance with CT abdomen with contrast was pursued. Representative abdominal imaging demonstrating cirrhotic liver morphology with portal hypertension without focal malignant lesions is shown in Figure 3.

In 2024, recurrent elevation of ferritin prompted resumption of therapeutic phlebotomy. Although iron depletion was effective, treatment intensity was limited by the development of anemia, necessitating individualized hemoglobin thresholds and periodic interruption. By early 2025, ferritin levels were consistently maintained below 100 ng/mL, allowing transition to a maintenance phlebotomy strategy.

**Figure 4:**
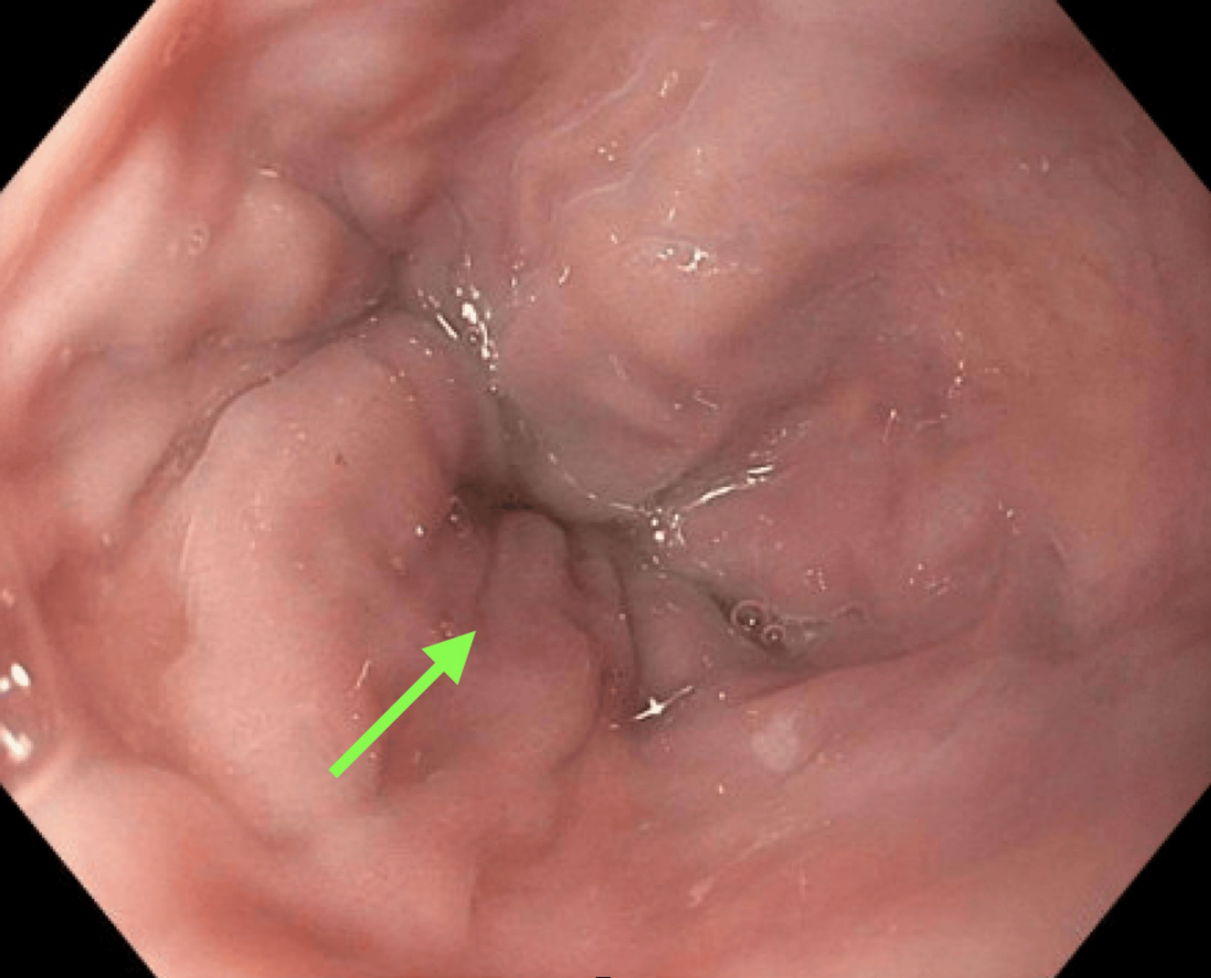
Upper Endoscopy Demonstrating Esophageal Varices Endoscopic image demonstrating grade I distal esophageal varices (green arrow) consistent with portal hypertension.

During subsequent follow-up, surveillance upper endoscopy demonstrated grade I distal esophageal varices, confirming clinically significant portal hypertension in the setting of advanced chronic liver disease (Figure 4). Despite established cirrhosis, the patient remained clinically stable without ascites or hepatic encephalopathy and continued immunosuppressive therapy for autoimmune hepatitis with preserved functional status.

## Discussion

Hereditary hemochromatosis is most commonly associated with homozygosity for the HFE C282Y mutation, which accounts for the majority of clinically significant iron overload cases in populations of Northern European ancestry [1]. In contrast, individuals who are heterozygous for the C282Y mutation are traditionally considered asymptomatic carriers, with large population studies demonstrating minimal penetrance and a low likelihood of developing clinically relevant iron accumulation or end-organ damage [2,3]. Accordingly, current screening and management guidelines generally do not recommend routine surveillance or therapeutic intervention for isolated C282Y heterozygosity in the absence of additional risk factors [4].

The present case is notable for the degree of iron overload observed in a C282Y heterozygous patient, with ferritin levels exceeding 3,800 ng/mL and histologically confirmed hepatic iron deposition with established cirrhosis. Such marked hyperferritinemia is distinctly unusual in simple heterozygotes and raises important diagnostic and pathophysiologic considerations. While mild elevations in serum ferritin may be seen in heterozygous carriers, levels of this magnitude are rarely attributable to HFE heterozygosity alone and should prompt evaluation for secondary contributors to iron dysregulation [5,6].

Chronic inflammatory and autoimmune liver diseases are well-recognized modifiers of iron metabolism. Autoimmune hepatitis, in particular, has been associated with elevated ferritin levels through a combination of inflammatory cytokine signaling, hepatocellular injury, and altered hepcidin regulation [7,8]. Inflammatory suppression of hepcidin can enhance intestinal iron absorption and promote parenchymal iron deposition, thereby unmasking or amplifying iron overload in genetically predisposed individuals [9]. In this patient, liver biopsy demonstrated both portal/interface hepatitis consistent with autoimmune hepatitis and moderate-to-marked iron deposition within hepatocytes and Kupffer cells, supporting a synergistic interaction between autoimmune liver disease and underlying genetic susceptibility (Figure 2).

Cirrhosis itself further complicates iron homeostasis. Advanced liver disease is associated with impaired hepcidin synthesis, increased iron absorption, and progressive hepatic iron accumulation, independent of HFE genotype [10]. The coexistence of cirrhosis and autoimmune hepatitis in this patient likely contributed to a feed-forward cycle of worsening iron overload, ultimately resulting in clinically significant hepatic iron deposition despite heterozygous HFE status [11]. This mechanism may explain why a subset of heterozygous individuals develop phenotypes typically reserved for homozygous disease.

Importantly, this case also demonstrates that therapeutic phlebotomy can be effective and well tolerated in such patients when true iron overload is confirmed. Following initiation of phlebotomy, the patient experienced a sustained reduction in ferritin levels to below 100 ng/mL, with maintenance therapy preventing reaccumulation of iron over time (Table 1). Although phlebotomy thresholds are traditionally derived from studies in C282Y homozygotes, emerging evidence suggests that selected heterozygous patients with documented iron overload and organ involvement may similarly benefit from iron depletion therapy [12,13]. Careful monitoring is required to avoid excessive anemia, particularly in patients with chronic inflammatory conditions or cirrhosis.

This case underscores several clinically relevant lessons. First, extreme hyperferritinemia in a C282Y heterozygote should not be dismissed as incidental or purely inflammatory without thorough evaluation, including histologic confirmation when appropriate. Second, autoimmune hepatitis and cirrhosis may act as critical modifiers that convert low-penetrance genotypes into clinically overt disease. Finally, individualized management strategies, including therapeutic phlebotomy, can lead to meaningful biochemical and clinical improvement even in non-classical presentations of hereditary hemochromatosis [14,15].

In summary, this report highlights a rare but important phenotype of severe iron overload and cirrhosis in a patient heterozygous for the HFE C282Y mutation. Recognition of modifying factors such as autoimmune liver disease is essential for timely diagnosis and appropriate treatment, and this case supports a more nuanced, phenotype-driven approach to iron overload syndromes beyond rigid genotype-based assumptions.

## Conclusions

This case demonstrates that clinically significant iron overload and cirrhosis may occur in HFE C282Y heterozygotes when additional modifiers are present, particularly autoimmune hepatitis, chronic hepatic inflammation, and cirrhosis-associated dysregulation of iron metabolism. Extreme elevations in ferritin should not be attributed solely to inflammation without evaluation for true iron overload. Histologic confirmation of hepatic iron deposition was pivotal in establishing the diagnosis and guiding management. Therapeutic phlebotomy resulted in sustained improvement in iron indices, supporting a phenotype-driven and individualized approach in selected heterozygous patients with documented iron overload. This case supports a phenotype-driven approach to iron overload syndromes, emphasizing that genetic heterozygosity does not preclude clinically meaningful disease. Increased awareness of this rare presentation may facilitate earlier diagnosis and appropriate treatment, ultimately improving long-term hepatic outcomes.
